# HLA-G/ILTs Targeted Solid Cancer Immunotherapy: Opportunities and Challenges

**DOI:** 10.3389/fimmu.2021.698677

**Published:** 2021-06-30

**Authors:** Aifen Lin, Wei-Hua Yan

**Affiliations:** ^1^ Biological Resource Center, Taizhou Hospital of Zhejiang Province, Wenzhou Medical University, Linhai, China; ^2^ Key Laboratory of Minimally Invasive Techniques & Rapid Rehabilitation of Digestive System Tumor of Zhejiang Province, Taizhou Hospital of Zhejiang Province, Linhai, China; ^3^ Medical Research Center, Taizhou Hospital of Zhejiang Province, Wenzhou Medical University, Linhai, China

**Keywords:** HLA-G, immune checkpoint, immune checkpoint inhibitor, immunoglobulin-like transcript, cancer immunotherapy

## Abstract

Immune checkpoint inhibitors (ICIs) have become a promising immunotherapy for cancers. Human leukocyte antigen-G (HLA-G), a neoantigen, its biological functions and clinical relevance have been extensively investigated in malignancies, and early clinical trials with “anti-HLA-G strategy” are being launched for advance solid cancer immunotherapy. The mechanism of HLA-G as a new ICI is that HLA-G can bind immune cell bearing inhibitory receptors, the immunoglobulin-like transcript (ILT)-2 and ILT-4. HLA-G/ILT-2/-4 (HLA-G/ILTs) signaling can drive comprehensive immune suppression, promote tumor growth and disease progression. Though clinical benefits could be expected with application of HLA-G antibodies to blockade the HLA-G/ILTs signaling in solid cancer immunotherapy, major challenges with the diversity of HLA-G isoforms, HLA-G/ILTs binding specificity, intra- and inter-tumor heterogeneity of HLA-G, lack of isoform-specific antibodies and validated assay protocols, which could dramatically affect the clinical efficacy. Clinical benefits of HLA-G-targeted solid cancer immunotherapy may be fluctuated or even premature unless major challenges are addressed.

## Introduction

Immune checkpoint inhibitors have become a promising immunotherapy for cancers, but durable clinical benefits are limited for existing agents (1). It’s an exciting news released in Cancer Discovery “Gilead Buys into Tizona’s Anti-HLA-G Strategy” that an early clinical trial with human leukocyte antigen-G (HLA-G) inhibitor TTX-080 is being launched for advance solid cancer patients (NCT04485013) (2).

HLA-G, firstly observed on extravillous cytotrophoblast, has been considered to play critical roles in maintaining maternal immune tolerance for the semi-allograft fetus during pregnancy (3, 4). In the context of malignancies, aberrant HLA-G expression in melanoma lesions but not adjacent normal tissues was reported by Paul and co-workers in 1998 for the first time (5). This pioneering investigation has been testified with thousands of samples in more than 30 types of cancers. Ever increasing studies on HLA-G expression by solid tumor lesions have revealed that high levels of HLA-G expression was associated with advanced disease stage, tumor metastasis, poor prognosis, or shorter disease-free survival. However, either among patients with different types of cancers, or among patients with the same type of cancer, intertumor and intratumor heterogeneity of HLA-G expression is evident, such as among patients with breast cancer (6–15), colorectal cancer (16–24), cervical cancer (25–27), endometrial cancer (28–31), esophageal squamous cell carcinoma (32–34), Ewing sarcoma (35), gastric cancer (36–38), glioblastoma (39), hepatocellular carcinoma (40–42), lung cancer (43–45), classical Hodgkin lymphoma (46, 47), diffuse large B-cell lymphoma (48), cutaneous T- and B-cell lymphoma (49), nasopharyngeal carcinoma (50), oral squamous cell carcinoma (51), ovarian cancer (52–55), pancreatic adenocarcinoma (56–59), and thyroid cancer (60, 61) (**Table 1**). HLA-G expression in solid cancers is now well acknowledged in promoting cancer cell immune escaping and tumor development, and associated with disease progression and poor survival either among cancer patients or pre-clinical murine models (62).

**Table 1:** HLA-G expression in solid cancers.

Cancers	Lesions	Method (Ab)	HLA-G (%)	Immuno-staining evaluation	Main findings	Ref.
Breast cancer	39	IHC (4H84)	41%	0, negative; 1–5% (1); 6–25% (2); 26–75% (3); 76–100% (4).	Associated with shorter disease-free survival.	(6)
	58	IHC (4H84)	70.7%	Negative (0); 1–25% (1); 26–50% (2); 51–75% (3); >75% (4).	Associated with advanced disease stage.	(7)
	235	IHC (HGY)	66%	Negative (−); <25% (+) and/or weakly; 25–50% and/or moderately (++); >50% and/or strongly stained (+++).	An independent prognosis factor.	(8)
	501	IHC (4H84)	60%	Positive, any staining of tumor cells; Negative, no staining.	A prognostic factor among classical HLA class I negative patients.	(9)
	52	IHC (5A6G7)	59.6%	Negative, <25% positivity; positive (>25% positivity.	Associated with aggressiveness.	(10)
	45	IHC(MEM-G/2)	62.2%	Positive, >15% of staining.	Associated with shorter survival.	(11)
	102	IHC (4H84)	94.1%	Negative (−); weak staining (+); moderate staining (++) and strong staining (+++).	HLA-G^low^ is associated with higher overall and relapse-free survival rates.	(12)
	73	HC (MEM-G/1)	43.8%	Positive, >25% of staining, irrespective of staining intensity.	Not associated with clinical parameters.	(13)
	2,042	IHC (4H84)	24%	Positive, any staining of tumor cells; Negative, no staining.	Not associated with clinical outcome.	(14)
	HER2+ (n = 28)	WB (4H84) (5A6G7)	HLA-G/GAPDH ratio	High and low levels of protein expression were determined by median.	Among HER2+ tumors, patients with HLA-G6 low had a higher pathological complete response.	(15)
Colorectal cancer	81	IHC		Based on presence or absence of positive stained cells.	HLA-G expressed in majority primary tumors but not in associated liver metastasis.	(16)
		(4H84)	29%			
		(MEMG/1)	35%			
		(MEM-G/2)	19%			
	201	IHC (HGY)	64.6%	Without staining (−); < 25% and/or weakly (+); 25–50% and/or moderately (++); > 50% and/or strongly stained (+++).	An independent prognosis factor.	(17)
	102	IHC (MEM-G/2)	70.6%	Based on presence or absence of positive stained cells.	Associated with worse survival.	(18)
	457	IHC (4H84)	70.7%	HLA-G positive when >5%, irrespective of staining intensity.	HLA-G expression >55% associated with worse prognosis.	(19)
	285	IHC (4H84)	22.1%	Intensity of staining (absent, weak, moderate, or strong).	Associated with worse survival and disease-free survival.	(20)
	484	IHC (4H84)	27.7%	Intensity of staining (absent or faint in <20%), weak (faint to weak in >20% but ≤70%), moderate (weak to moderate in >70%), or strong (intense in 20~70%).	Associated with presence of the Foxp3+ cells.	(21)
	88	IHC (4H84)	59.1%	Total score of the proportion and intensity scores for negative and positive tumor cell (ranges = 0–9). Cut point scores for positive and negative tumor cells are ≥4.	Increased expression of HLA-G correlated with tumor node metastasis staging.	(22)
	379	IHC		The percentage of HLA-G positive tumor cells based on presence of HLA-G staining while irrespective the staining intensity. HLA-G >5% in a section was considered as positive. Difference of the percentage of HLA-G positive tumor cells (ΔHLA-G) in the case-matched CRC samples was calculated by the percentage of HLA-G detected with mAb 4H84 subtracted that with mAb 5A6G7. According to value of ΔHLA-G, three groups were obtained: ΔHLA-Gneg (ΔHLA-G> −5.0%), ΔHLA-Gcom (−5.0%≤ΔHLA-G ≤ 5.0%), and ΔHLA-Gpos (ΔHLA-G>5.0%).	HLA-Gneg in 64(16.9%), ΔHLA-Gcom in 159 (42.0%), and ΔHLA-Gpos in 156 (41.2%), mAbs 4H84neg5A6G7pos in 44 (11.6) CRC cases was observed. Both ΔHLA-G and its subgroups mAbs 4H84^neg^5A6G7^pos^ and 4H84 pos5A6G7 neg status were significantly related to survival.	(23)
		(4H84)	70.7%			
		(5A6G7)	60.4%			

	157	Flow cytometry (MEM-G/09)	Median of HLA-G:14.9% (range: 1.8–80.0%)	Among EpCMA+ colorectal tumor cells.	Higher HLA-G percentage associated with patient poor survival.	(24)
Cervical cancer	58	IHC (5A6G7)	75.86%	No expression (0); 1–30% (1); 31–70% (2); 71–100% positive cells (3).	An early marker for progression.	(25)
	143	IHC (4H84)	60%	Membrane or combined membrane and cytoplasmic expression of HLA-G were interpreted as positive.	Associated with disease progression.	(26)
	79	IHC (5A6G7)	31.6%	Low expression when no signal or discrete staining; high expression when moderate or intense staining.	HLA-G detected in 17 (32.7%) without and 8 (29.6%) with metastasis.	(27)
Endometrial carcinoma	44	IHC(4H84)	55%	Negative (0); 1–5% (1); 6–10% (2); 11–25% (3); 26–50% (4); >50% (5).	Associated with disease stage.	(28)
	525	IHC (4H84)	39.8%	Negative (0); 1–5% (1); 5–25% (2); 25–50% (3); 50–75% (4), and >75% (5). The intensity scored 0: absent, 1: weak, 2: moderate, 3: strong. The sum of both scores. A score of ≥2.5 considered as up-regulation of HLA-G.	Not associated with survival.	(29)
	40	IHC (4H84)	40%	Both membrane-bound and cytoplasmic HLA-G expression were considered as positive.	Not associated with survival.	(30)
	113	WB (MEMG/1)	HLA-G/GAPDH ratio	High and low levels of protein expression were determined by median.	Higher levels of HLA-G 56 kDa isoforms were observed in patients with metastases to lymph nodes	(31)
Esophageal cancer	121	IHC (HGY)	90.9%	Without staining(−); <25% and weakly (+); 25~50% and moderately (++); >50% and strongly stained (+++).	An independent prognosis factor.	(32)
	79	IHC (4H84)	65.8%	HLA-G expression was graded as: negative, 1~25% (1+), 26~50% (2+), 51~75% (3+), and >75% (4+), irrespective of staining intensity.	HLA-G is an independent prognosis factor.	(33)
	60	IHC (MEM-G/1)	70%	Without staining (0); <25% (1+); 25~50% (2+); and >50% (3+). Negative and 1+ as HLA-G negative, 2+ and 3+ as HLA-G positive.	Associated with cancer cell differentiation, lymph node metastasis.	(34)
Ewing sarcomas	47 (primary)	IHC (4H84)	30%	Graded by low, intermediate or strong densities.	Associated with tumor infiltrating T cells.	(35)
	12 (relapse)		33%			
Gastric cancer	160	IHC (HGY)	71%	Without staining (−); <25% and/or weakly (+); with 25~50% and/or moderately (++); >50% of the cancer tissues and/or strongly stained (+++).	An independent prognosis factor.	(36)
	52	IHC (5A6G7)	31.%	HLA-G positivity when >10%.	An independent prognosis factor.	(37)
	179	IHC (4H84)	49.7%	Negative; 1~25% (+); 25~50% (++); >50% (+++).	An independent prognosis factor.	(38)
Glioblastoma	108	IHC (MEM-G/2)	60.2%	No details described.	HLA-G-negative patients were alive longer than HLA-G positive patients.	(39)
Hepatocellular carcinoma	173	IHC (MEM-G/1)	low (43%)	The density of HLA-G staining evaluated with computerized image system.	Associated with poor survival and increased recurrence;	(40)
			high (57%)			
	36	WB (MEM-G/1)	66.7%	No details described.	An independent prognosis factor.	(41)
	219	IHC (4H84)	50.2%	Negative, and positive grouped as 1~25%, 26~50%, 51~75%, and >75%.	Associated with advanced disease stage.	(42)
Lung cancer	106	IHC (HGY)	75%	Without staining (−); <25% and weakly (+); 25~50% and moderately (++); >50% and strongly stained (+++).	An independent prognosis factor.	(43)
	101	IHC (4H84)	41.6%	Negative (0), 1~25% (1), 26~50% (2), and >50% (3), irrespective of staining intensity.	Associated with advanced disease stage.	(44)
	131	IHC (5A6G7)	34%	Negative ≤ 5% and positive >5%.	Predominately expressed in adenocarcinoma.	(45)
Lymphoma (classical Hodgkin)	175	IHC (MEM-G/1)	54%	Positive when >50% of neoplastic cells showed stronger staining.	Associated with absence of MHC class I expression on HRS cells and EBV negative status.	(46)
	20	IHC (4H84)	55%	Negative staining (0), <25% (1), 26~50% (2), 51~75% (3), 76~100% (4).	Different patterns of HLA-G expression associated with different outcomes.	(47)
Lymphomas (Diffuse Large B-Cell)	148	IHC (4H84)	24%	Positive when >25% of lymphoma cells expressed intermediate/strong staining.	Negative HLA-G expression associated with worse survival.	(48)
Lymphomas (cutaneous T- and B-cell)	45	IHC (4H84)	51%	HLA-G positivity as a strong (numerous cells) or as a single-cell positivity (scant, scattered cells throughout the infiltrate).	Associated with high-grade histology and advanced stage in CTCL.	(49)
Nasopharyngeal carcinoma	552	IHC (4H84)	79.2%	Intensity as (neg); weak (1); moderate (2); strong (3). Percentage <5% (0); 5–25% (1); 26–50% (2); 51–75% (3); 76–100% (4). A score by adding intensity and positive cells.	Associated with poor prognosis, disease recurrence or metastasis.	(50)
Oral squamous cell carcinoma	60	IHC (MEM-G/2)	50%	An immunoreactive score (IRS) calculated by multiplying the percentage and staining intensity. IRS = 0 (negative); <2 (low); >2 (high).	Lower HLA-G expression associated with longer survival.	(51)
Ovarian cancer	40	IHC (4H84)	Low (55%)	0–25% stained tumors and mild staining (1+); 25–50% and moderately staining (2+); >50% and strongly staining (3+).	HLA-G expression >17% associated with poor survival.	(52)
			moderate (20%)			
			strong (25%)			
	34	IHC (MEM-G/2)	35%	No details described.	Associated with high-grade histology.	(53)
	118	IHC (5A6G7)	79.7%	Percentage of stained cells >5% (+); <5% (−).	Not associated with clinical parameters.	(54)
	62	IHC (4H84)	72.4%	The scores correspond to the percentage of positive tumor cells of <1% (score 0); 1–5% (score 1), 6–25% (score 2), 26–50% (score 3), and >50% (score 4). Score 1 and score 2 were considered as “low positive percentage cells” scores. Whereas, score 3 and score 4 were considered as “high positive percentage cells” scores.	Positive HLA-G expression was highly represented in patients with ovarian carcinoma recurrence.	(55)
Pancreatic adenocarcinoma	122	IHC (Rabbit polyclonal)	low (36.1%)	0, none; 1, ≤25%; 2, 26~50%; 3, >50%). Intensity (0, none; 1, weak; 2, moderate; 3, strong).	An independent prognosis factor.	(56)
			high (63.9%)			
	42	IHC (4H84)	66%	1–25% (negative), 26–50%, 51~75%, and >75%, irrespective of staining intensity.	Associated with advanced stages	(57)
	158	IHC (not described)	39.2%	Negative: <5%; local: 5–75%; diffuse: >75%, irrespective of staining intensity.	Associated with worse survival.	(58)
	243	IHC (4H84)	36.7%	Strongly (+++), with almost all cancer cells (≥90%) staining strongly; moderately (++), with <90 and ≥50% of cancer cells staining strongly; weakly positive (+), with <50 and >10% of cancer cells staining strongly or >5% of cancer cells staining weakly; negative (−), with ≤5% of cancer cells staining.	High HLA-G associated with both shorter overall survival and disease-free survival.	(59)
Thyroid carcinoma	138	IHC (5A6G7)	90.6%	Without staining (–); <25% (+); 25~50% (++); >50% of cell staining (+++).	Associated with poor prognosis	(60)
	70	IHC (MEM-G/2)	44.3%	No details described.	Associated with lymph node metastasis.	(61)

**Figure 1:**
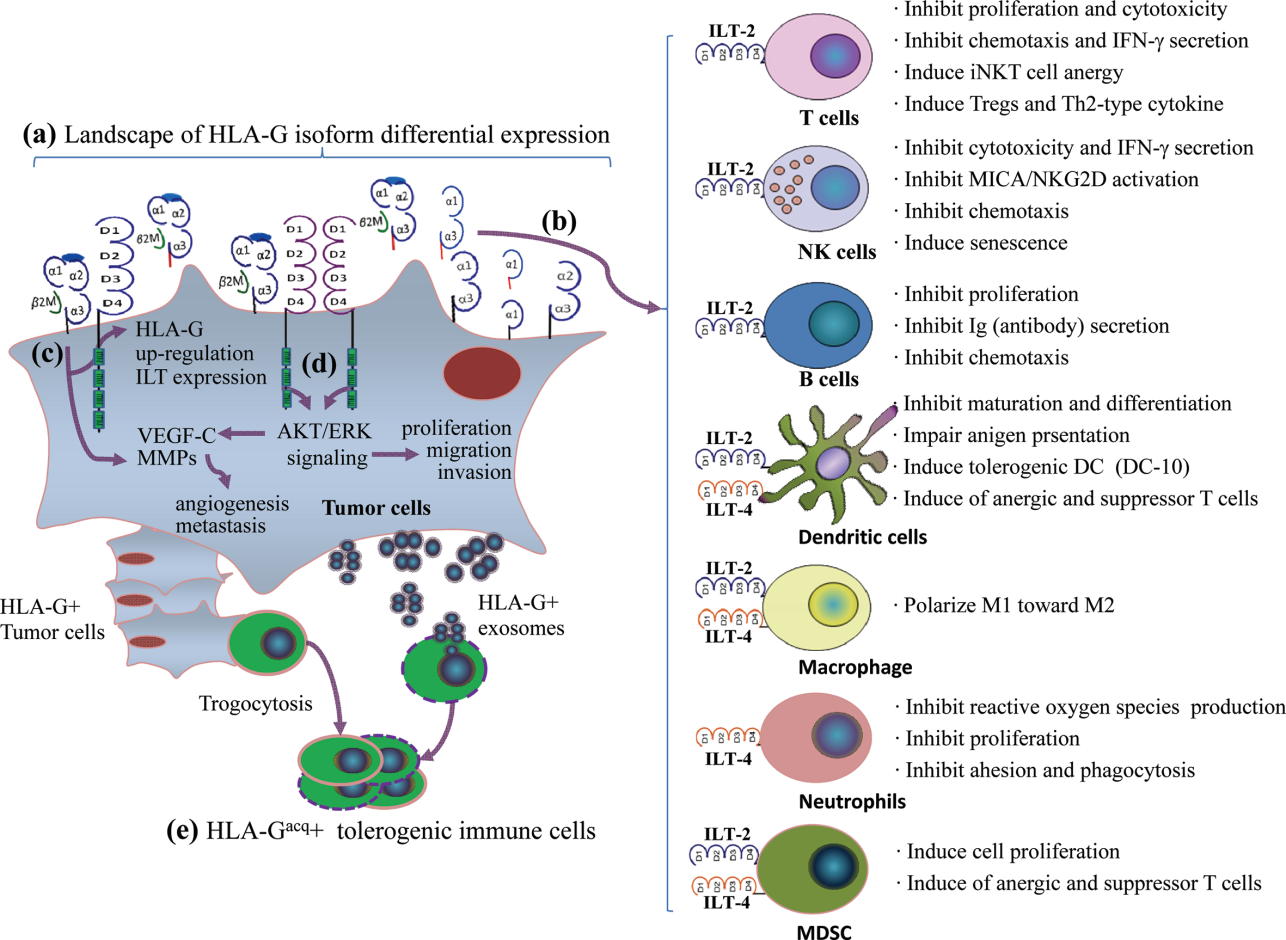
A comprehensive immune suppression mediated by HLA-G/ILTs engagement in cancer development. **(A)** Distinct profiles of HLA-G isoform expression in an individual cancer patient. The heterogeneous landscape of HLA-G isoform differential expression among cancer patients can be temporal and spatial dependent. **(B)** Immune inhibitory receptors ILT-2 and ILT-4 are expressed on different immune cell. ILT-2 recognizes HLA-G1 and HLA-G5 while ILT-4 recognizes HLA-G1, -G2, -G5, and HLA-G6 isoforms. HLA-G isoform-dependent ILT-2 and ILT-4 engagement induces a wide spectrum of immune suppression which benefits cancer cell escaping from host immune surveillance and anti-tumor immunity. **(C)** HLA-G expression up-regulates intratumor ILTs and MMPs expression. **(D)** ILTs induce VEGF-C expression and enhance cancer cell proliferation, migration and invasion through AKT/ERK signaling, which favors cancer cell angiogenesis and metastasis. **(E)** In addition to direct binding between HLA-G and ILTs, immune cells acquire HLA-G from neighboring HLA-G+ cancer cells through contact-dependent trogocytosis and from cancer cell derived HLA-G-bearing exosomes in a long-distance. HLA-G acquired immune cells became tolerogenic phenotype and immune functions are impaired.

Engagement of HLA-G/ILTs can induce either unresponsive or tolerogenic state of a wide range of immune effector cells. The underlying mechanisms for HLA-G as an immune checkpoint is that HLA-G can either directly bind tyrosine-based inhibitory motifs (ITIMs) containing immune inhibitory receptors, the immunoglobulin-like transcript (ILT)-2/CD85j/LILRB1 and ILT-4/CD85d/LILRB2, which is expressed on various immune competent cells such as T lymphocytes, natural killer cells (NK), dendritic cells (DC) (62), or indirectly by intercellular transfer by the process of trogocytosis and exosomes to drive a comprehensive immune suppression (63). Immune suppression induced by the HLA-G/ILTs signaling pathway includes inhibition of cytotoxicity (64), inflammatory cytokine production (65), chemotaxis and proliferation of T cells and NK cells (66, 67), inhibition antibody production of B cells and maturation of antigen presenting cells (68, 69), and dampen the anti-tumor functions of invariant natural killer T (iNKT) cells and tumor-infiltrating CD8^+^PD-1^−^ILT-2^+^ T cells (70, 71). Also, HLA-G/ILTs engagement can also induce expansion of myeloid derived suppressive cells (MDSCs) and generation of regulatory T cells (72, 73). In addition to immune suppressive functions, HLA-G/ILTs can promote intratumor vascular remodeling by enhancing vascular endothelial growth factor-C (VEGF-C) expression, and increase tumor metastasis by inducing cancer promoting factor matrix metalloproteinases (MMPs) expression (74, 75) (**Figure 1**). Consequently, both innate and adaptive antitumor immune responses are impaired, thus favoring tumor cell immune evasion and disease progression. In this scenario, restoring antitumor functions of ILT-bearing immune cells with HLA-G inhibitors such as TTX-080 sounds reasonable.

As HLA-G expression is specifically induced in most types of solid cancer cells, clinical benefits of HLA-G inhibitors could be expected for cancer immunotherapy. However, challenges such as multiple HLA-G isoforms with distinct extracellular domains, different binding sites between HLA-G and ILT-2/ILT-4 interaction, intratumor or intertumor heterogeneity of HLA-G expression, and isoform-specific antibody and validated assay protocol lacking, remain tremendous hurdle in terms of HLA-G/ILTs antibody-based solid cancer immunotherapy.

## HLA-G Isoforms Molecular Structure

The *HLA-G* gene contains eight exons and seven introns. However, most full-length transcripts carry only seven exons because exon seven is usually spliced out. Due to a premature stop codon in E6, the HLA-G full-length protein has 338-amino acids, which is relatively shorter compared with classical HLA class I molecules. Among these exons, E1 generates the signal peptide, E2-E4 generate extracellular α1, α2, and α3 domains, respectively. E5 generates the transmembrane domain, and E6 generates the intracellular cytoplasmic tail of HLA-G (76).

**Figure 2:**
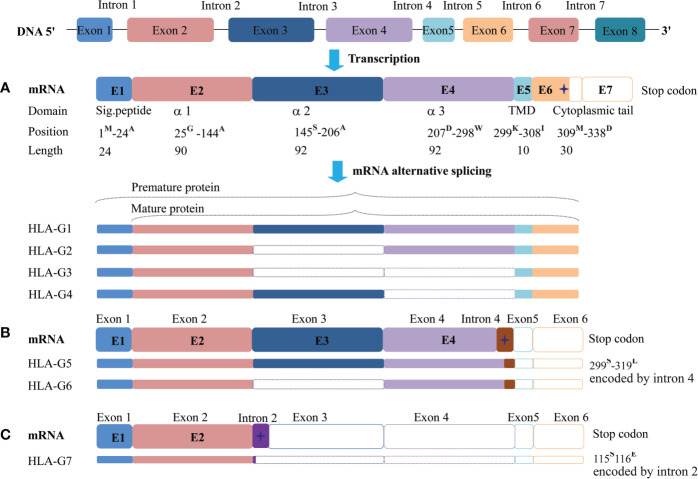
Seven identified HLA-G isoforms generated from its primary transcript alternative splicing. **(A)** The heavy chain of membrane-bound isoforms HLA-G1, -G2, -G3, -G4 generated by an mRNA containing a stop codon in exon 6. **(B)** Soluble isoforms HLA-G5 and HLA-G6 generated by an mRNA with a pre-stop codon in intron 4, which terminates transmembrane and cytoplasmic tail transcription. **(C)** Soluble isoforms HLA-G7 generated by an mRNA with a pre-stop codon in intron 2, which terminates the following domain transcription. Sig.peptide, Signal peptide; TMD, transmembrane domain; 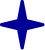, stop codon. The superscript capital letter represents amino acid at the position.

Due to its primary transcript alternative splicing, diverse molecular structures of HLA-G have been observed. Seven HLA-G isoforms including four membrane-bound (HLA-G1–HLA-G4) and three soluble (HLA-G5–HLA-G7) monomers have been identified. With a premature stop codon in E6, membrane-bound (HLA-G1–HLA-G4) isoforms have a unique truncated cytoplasmic tail comparing to other classic HLA class I molecules. Soluble HLA-G5 and HLA-G6 isoforms are resulted from a stop codon in intron 4, and HLA-G7 are generated from a stop codon in intron 2, which prevents the translation of their transmembrane domain (77, 78) (**Figure 2**).

**Figure 3:**
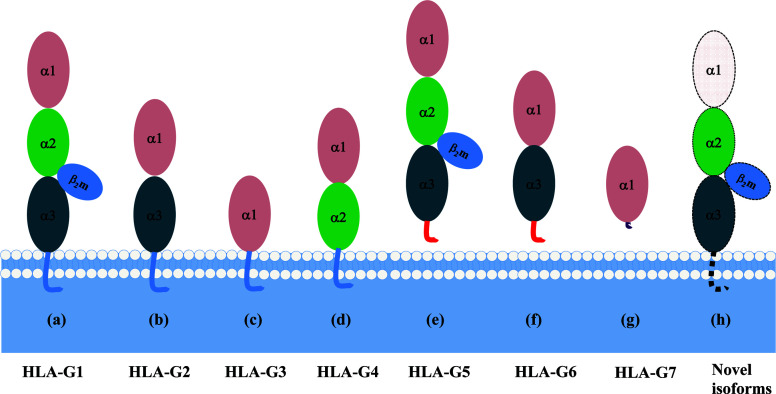
A schematic structure of HLA-G isoforms. **(A)** HLA-G1 have α1, α2, and α3 extracellular domains; **(B)** HLA-G2 have α1, and α3 extracellular domains; **(C)** HLA-G3 have α1 extracellular domains; **(D)** HLA-G4 have α1, and α2 extracellular domains; **(E)** HLA-G5 have α1, α2, and α3 extracellular domains; **(F)** HLA-G6 have α1 and α3 extracellular domains; **(G)** HLA-G7 has α1 extracellular domain followed by two C- terminal amino-acids encoded by intron 2; **(H)** Novel HLA-G isoforms such as lacking a transmembrane region and α1 domain have been predicted, but their structure remains confirmed.

Each HLA-G isoform has its unique extracellular structure. HLA-G1 is the only full-length isoform with extracellular α1, α2, and α3 domains; HLA-G2 has α1 and α3 domains; HLA-G3 has the only α1 domains; HLA-G4 has α1 and α2 domains. Similarly, HLA-G5 has the extracellular α1, α2, and α3 domains; HLA-G6 has α1 and α3 domains, and HLA-G7 has the only α1 domains. α1 and α2 domains form the peptide binding cleft, and α3 domain non-covalently bind to the light chain β_2_-microglobulin (β_2_m). Novel HLA-G isoforms such as lacking a transmembrane region and α1 domain have been predicted with RNAseq technology (79) (**Figure 3**). Moreover, higher molecular weight of HLA-G has been associated with post-translational modifications. Homo- and hetero-HLA-G dimers can be formed through intermolecular disulfide bonds with Cys^42^ or Cys^147^ in the extracellular α1 or α2 domain; others such as glycosylated, nitrated, and ubiquitinated HLA-G molecules have also been confirmed (80–83).

## HLA-G/ILTs Binding

ILT-2 and ILT-4 belong to the type I transmembrane glycoproteins, which have four extracellular immunoglobulin-like domains (D1-D4), a transmembrane region, and an intracellular tail with four or three immunoreceptor tyrosine-based inhibitory motifs (ITIMs). ILT-2 can be found on a variety of immune cells, such as subpopulations of T cells, B cells, natural killer (NK) cells, myeloid-derived suppressive cells (MDSCs), dendritic cells (DCs), and monocytes/macrophages. ILT-4 is not expressed on lymphocytes, but on monocytes/macrophages, neutrophils, basophils, DCs, and MDSCs (84, 85).

**Figure 4:**
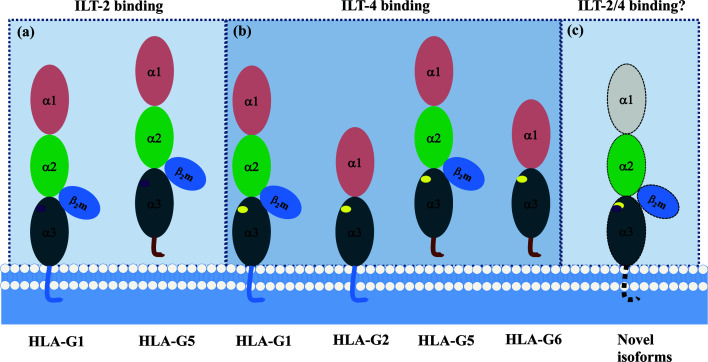
Different binding sites between HLA-G/ILT-2 and HLA-G/ILT-4. ILT-2 residues Tyr38 and Tyr76 bind Phe195 in HLA-G α3 domain, ILT-4 residues Tyr36 and Arg38 bind Phe195–Tyr197 loop in HLA-G α3 domain. **(A)** ILT-2 binds HLA-G heavy chain associated with β_2_m (HLA-G1 and HLA-G5). **(B)** ILT-4 binds both β_2_m-free (HLA-G2 and HLA-G6) and β_2_m-associated (HLA-G1 and HLA-G5) HLA-G heavy chain. **(C)** A panel of novel HLA-G isoforms including isoforms without α1 domain and transmembrane region, or with an extended 5’-region generated by HLA-G mRNA alternative splicing were predicted. However, molecular structure of these novel isoforms and remain to be identified, and interaction with ILTs is unknown yet. 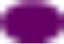 and 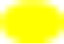 represent ILT-2 and ILT-4 binding site in HLA-G isoforms.

A recent study revealed that ILT-2/-4 extracellular D1D2 are responsible for the interaction with HLA-G binding, while D3D4 act as a scaffold (86). Also, ILT-2/-4 are more accessible to the HLA-G dimer binding than that of HLA-G monomer, leading to much stronger inhibitory signals. ILT-2 and ILT-4 extracellular D1D2 binds to the extracellular α3 domain of HLA-G, but structurally dependents. ILT-2 only binds to the HLA-G heavy chain associated with β_2_m, while ILT-4 can bind to both β_2_m free HLA-G heavy chain and HLA-G heavy chain with β_2_m. Moreover, residues Tyr^38^ and Tyr^76^ in ILT-2 are responsible for binding to HLA-G Phe^195^ in the α3 domain, whereas residues Tyr^36^ and Arg^38^ in ILT-4 interact with the Phe^195^–Tyr^197^ loop in the HLA-G α3 domain (87). The different binding sites between ILT-2/-4 and HLA-G could be an explanation for the higher affinity of ILT-4 than the affinity of ILT-2 when they interact with HLA-G (88). Based on the different extracellular structure of HLA-G isoforms and ILT binding characteristics, ILT2 can bind to the β_2_m associated HLA-G1 and HLA-G5 isoforms. However, ILT-4 can bind to both β_2_m associated or β_2_m free HLA-G isoforms, which include HLA-G1, -G2, -G4, -G5 and HLA-G6 isoforms (**Figure 4**).

Taking advantage of the early studies on fetal-maternal immune tolerance, HLA-G-mediated immune inhibition has been well-acknowledged in the broader spectrum of health and disease situations. HLA-G expression favors the acceptance of allograft organ transplantation, whereas it provides an additional strategy for cancer cells to escape from immune surveillance and clearance (89, 90). HLA-G/ILTs engagement activates the phosphorylation of the ITIMs contained in the tyrosine residues, which present docking sites for Src homology 2 (SH2) protein tyrosine phosphates SHP-1 and SHP-2, thus initiating an inhibitory signaling cascade. Meanwhile, ITIM-dependent recruitment of SHP1/SHP2 can markedly suppress the ITAM activated Syk/Src signal cascades in immune cell activation (91).

## Challenges in HLA-G/ILTs Targeted Solid Cancer Immunotherapy

One strategy successfully deployed by cancer cells for immune evasion is the impairment of the classical HLA class I and II antigens to hide infected cells from T cell recognition, while aberrant induction of HLA-G expression by cancer cells makes host anti-tumor immune system rather vulnerable (92). Though development of HLA-G/ILTs interaction targeted ICIs is promising for cancer immunotherapy, much real-world information on HLA-G/ILTs status is extremely necessary for both future basic and clinical investigations.

### Lack of HLA-G Isoform-Specific Monoclonal Antibodies

The most fundamental task is to develop HLA-G molecule universal or distinct HLA-G isoform-specific mAbs. As high as 80% amino acid sequence identity sharing in the extracellular domain of all HLA class I antigens, only distinct feature of HLA-G is its molecular weight of 39 kDa (HLA-G1 isoform) which is less than 45 kDa of the other classical HLA class I antigens (93), and this is rather similar to other non-classical HLA class I antigens including HLA-E and HLA-F (https://www.uniprot.org/uniprot/P17693; https://www.uniprot.org/uniprot/P13747; https://www.uniprot.org/uniprot/P30511). CLUSTALO sequence alignment results showed that, among HLA-G, HLA-F, and HLA-E molecules, the full-length amino acid sequence are identity round 62%, 75.9% between HLA-G and HLA-F, 71.2% between HLA-G and HLA-E, and 40.1% between HLA-E and HLA-F (**Supplementary Figure 1**). Thus, the cross-reactivity of anti-HLA-G mAbs to other classical and non-classical HLA class I antigens remains a huge task to be improved (94). Indeed, cross-reactivity of the most widely used such as mAb 4H84 for denatured HLA-G form has been observed to react with other HLA class I antigens (95, 96). Moreover, due to *HLA-G* primary transcript alternative splicing, in addition to seven ever-identified HLA-G isoforms and more novel isoforms can be expected, lack of HLA-G isoform-specific mAb prevents advances in characterizing their biological functions and clinical significance (97). To be noted, a panel of novel HLA-G isoforms predicted by deep transcriptome analysis have been reported. Among these isoforms, HLA-G1L have extra five amino acids (NKTPR) ahead the beginning residue Methionine at the N-terminal ends, others such as isoforms lack α1 or both α1 and α2 extracellular domains, and novel soluble HLA-G isoforms with distinct C-terminal ends generated by skipping exons 5 and 6.

**Figure 5:**
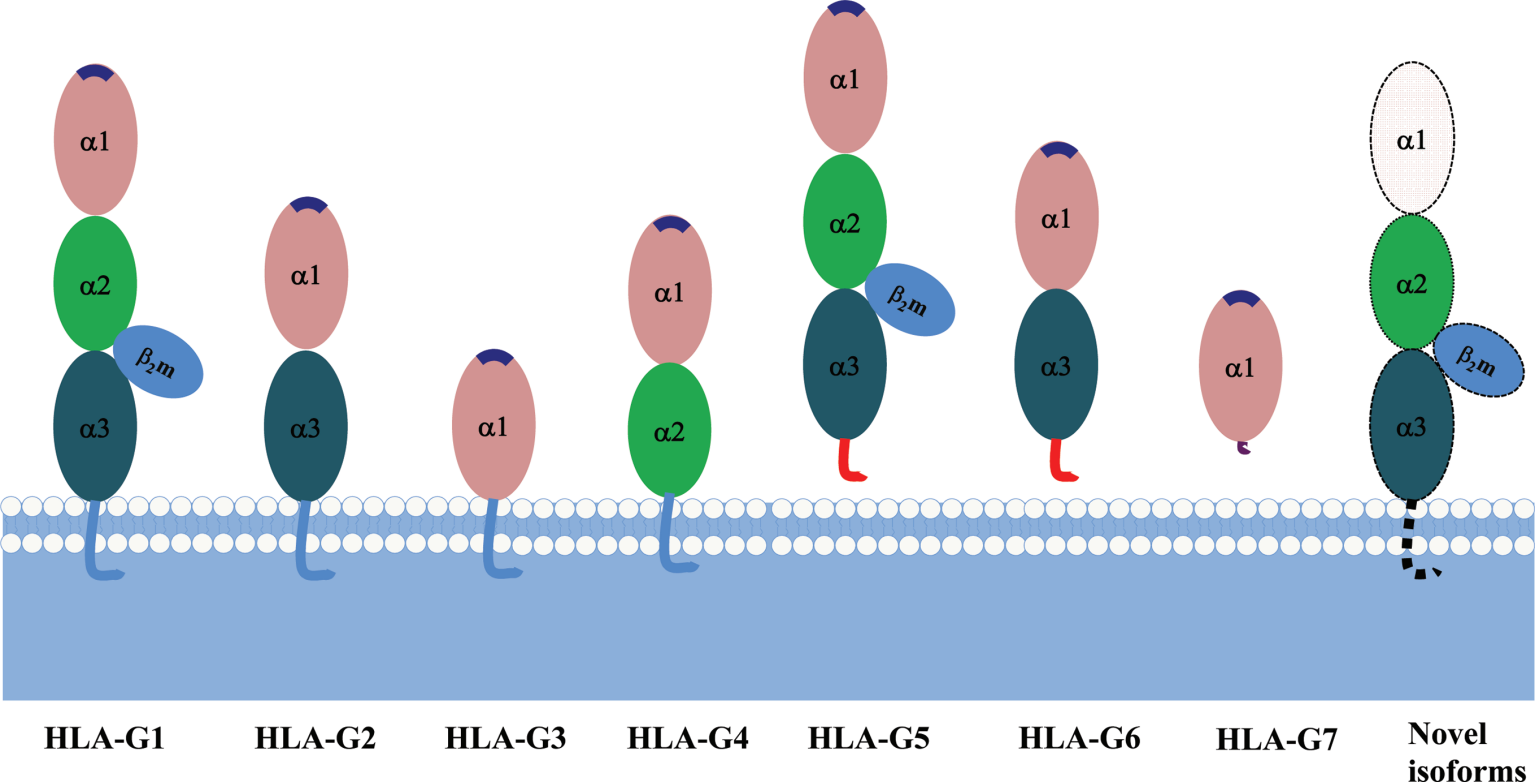
Epitopes in HLA-G recognized by mAbs 4H84 and 5A6G7. Among HLA-G antibodies, only mAbs 4H84 and 5A6G7 were generated with definite immunogen epitopes. mAb 4H84 generated by 61^st^~83^rd^ amino acids (EEETRNTKAHAQTDRMNLQTLRG) in HLA-G α1 domain which recognizes denatured heavy chain of all seven identified HLA-G isoforms containing α1 domain. mAb 5A6G7 generated by 21-mer C-terminal amino acid (SKEGDGGIMSVRESRSLSEDL) in HLA-G5 and -G6 isoforms, which recognizes both native and denatured heavy chain of HLA-G5 and HLA-G6 isoforms. Novel HLA-G isoforms such as isoforms without α1 domain and transmembrane region, or with an extended 5’-region were predicted. However, no current antibody is available to detect. 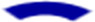 and 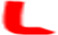 represent mAb 4H84 and 5A6G7 recognizing site in HLA-G isoforms.

Among anti-HLA-G antibodies, mAbs 4H84 and 5A6G7 are with known recognizing epitope in HLA-G heavy chain. mAb 4H84 generated by amino acid residues from 61 to 83 (in α1 domain HLA-G, which probes all denatured HLA-G isoforms (98). mAb 5A6G7 generated by a 22-mer C-terminal amino acid sequence in HLA-G5 and HLA-G6, which probes both native and denatured HLA-G5 and HLA-G6 isoforms (99, 100). mAbs 4H84 and 5A6G7 are the most widely used antibodies for evaluating total HLA-G (mAbs 4H84 and MEM-G/1) or HLA-G5/6 isoforms (mAb 5A6G7) respectively in malignant lesions with immunohistochemistry. As a result, novel isoforms without α1 domain can’t be detected by the mAb 4H84 (23, 79). In this scenario, the interpretation of clinical significance of HLA-G in cancers seems rather premature unless more reliable specific anti-HLA-G mAbs are used (**Figure 5**).

Furthermore, evaluation criteria for lesion HLA-G expression including staining protocols, cut-off levels, and cross-assay concordance are far from standardized, which could dramatically affect the definition of HLA-G expression and interpretation of its clinical significance, even with a same mAb to detect HLA-G within a certain type of cancer (62, 96). In this context, a reference criterion should be recommended by international community for HLA-G staining to minimize discrepancies across studies is urgently warranted.

Also, given different binding specificity of HLA-G between ILT-2 and ILT-4 resulted from its extracellular structure, that ILT-2 binds HLA-G1 and HLA-G5 while ILT-4 binds HLA-G1, -G2, -G4, -G5, and HLA-G6 isoforms, the landscape of HLA-G isoforms and their degree of expression in cancers can tremendously affect the benefits of HLA-G/ILTs based cancer immunotherapy.

### Heterogeneity of HLA-G Expression in Malignancies

Accumulating evidence solidify the concept that tumor heterogeneity, including inter-patient, inter-tumor, and intra-tumor heterogeneity, is the main cause for variable responses and clinical outcomes to anti-cancer therapy (101). Since the first report HLA-G expressed in tumors, the degree or proportion of HLA-G expression in thousands of malignant lesions among over thirty different types of cancers have been explored. Data revealed that HLA-G expression is restricted to malignant lesions, but not in adjacent non-tumorous tissues, and that neo-HLA-G expression is strongly related to metastasis, advanced disease stage, poor prognosis, and clinical outcome (89). However, inter-patient, inter-tumor, and intra-tumor heterogeneity of the HLA-G expression in each histopathological type of malignancies is also evident (102, 103). In addition to the HLA-G heterogeneity caused by tumor cell itself, such as clonal growth with genetic alterations, epigenetic and post-translational modifications, explanation of HLA-G expression could be biased due to usage of different current available anti-HLA-G monoclonal antibodies, and different assay protocols (62, 96). Very recently, using the method of flow cytometry, our data showed that HLA-G expression in 157 epithelial cell adhesion molecule (EpCAM) positive-gated colorectal tumor lesions is with a median of 14.90% (range: 1.81~79.90%) (24).

For an example, the inter-patient proportion of HLA-G expression in cancers has been observed from 24 to 94.1% in breast cancers (12, 14), and from 22.1 to 70.7% in colorectal cancers (19, 20). Noteworthy, only few studies on the intrapatient inter-tumor and intra-tumor heterogeneity of the HLA-G expression were available. In a cohort of 136 primary cervical cancers, inter-tumor heterogeneity of the HLA-G expression was detected in 25% of these lesions and 11% of case-matched lymph node (LN) metastases. Among pathological subtypes, HLA-G was positive in 22% in squamous cell carcinoma lesions and 20% in LN metastatic tissues, 31% in adenocarcinoma lesions, and 28.0% in their LN metastatic samples. In patients with invasive cervical cancer, positive proportion of HLA-G expression was found in 31.6 and 29.6% of the primary and LN metastatic lesions, respectively (104). Similarly, a study by Swets et al. (16) showed that HLA-G expression was found in 29% of the primary colorectal cancer and 30% of the corresponding liver metastases. Finally, regarding the intra-tumor heterogeneity of the HLA-G, Rouas-Freiss et al. (103) reported that, among 19 clear cell renal-cell carcinoma lesions, the proportion of HLA-G expression varies dramatically on CA9+ clear cell renal-cell carcinoma cells in different zones in each sample, which could be ranged from negative to almost totally positive for HLA-G expression. Also with clear cell renal-cell carcinoma lesions, a study by Tronik-Le Roux et al. (79) released that HLA-G isoform expression including HLA-G1, -G5, and HLA-G6 are extremely heterogeneous among distinct subcellular locations and zones within a same tumor. In a serial section study with colorectal and esophageal cancer lesions, our recent findings further revealed that intratumor heterogeneous expression of HLA-G is a very frequent phenomenon among different zones within a tumor (102).

### Native HLA-G Isoform Expression in Cancer Lesions Needs Evaluated

As aforementioned, most currently available information of HLA-G expression in cancer lesions were evaluated by immunohistochemistry with the mAbs 4H84 and/or 5A6G7, which represents all α1 domain containing HLA-G or HLA-G5/6 isoform expression. Whether these data are consistent with the levels of tumor cell surface HLA-G expression remain elusive. Previous evidence showed that no correlation has been established between the degree of cancer lesion HLA-G5/6 expression evaluated with immunohistochemistry and peripheral soluble HLA-G levels (42). Given the fact that HLA-G/ILTs interaction is conformation dependent, tumor cell surface native or conformational HLA-G expression be evaluated with assays such as flow cytometry is necessary (105). To address this issue, more reliable and specific anti-native or -conformational HLA-G mAbs are yet to be explored.

## Other HLA-G Receptors

In addition to the ILT-2 and ILT4, receptors including killer inhibitory receptor (KIR) 2DL4/CD158d, CD8, CD160 could also bind HLA-G (106). To be noted, NKG2A/CD94 has been recently reported which could bind to allelic specific products of HLA-G (107).

KIR2DL4 is a member of the killer cell immunoglobulin (Ig)-like receptor (KIR) family, but with an atypical feature owing to a D0 and D2 hybrid extracellular domain, a positively charged arginine residue in the transmembrane region and one ITIM domain in its cytoplasmatic tail. The charged arginine residue enables KIR2DL4 to associate with the Fc fragment receptor γ (FcRγ), which contains two cytoplasmic immunoreceptor tyrosine-based activation motifs (ITAMs). With this unique structure, KIR2DL4 is of both the activation (ITAM) and inhibitory (ITIM) signaling domains (108). The biological function of KIR2DL4 and HLA-G binding is to work as an activating receptor, which induces strong pro-inflammatory cytokine and chemokine immune responses through the endosomal DNA-dependent protein kinase (DNA-PKcs) signaling pathway, but not the NK cell cytotoxicity (109). KIR2DL4 is predominately expressed in decidual NK cells and HLA-G/KIR2DL4 interaction plays critical roles in the regulation of maternal-fetal immune microenvironment, spiralartery remodeling and fetal growth (4).

The heterodimer NKG2A/CD94, a member of the C-type lectin-like receptor family, is a well-known immune inhibitory receptor for HLA-E binding. NKG2A/CD94 is mainly expressed on CD8+ T lymphocytes and subsets of NK cells (110). Additionally, NKG2A/CD94 has recently been found to be an HLA-G allelic product dependent receptor. Hò et al. (107) revealed that a remarkably higher binding affinity was observed for the HLA-G*01:04/NKG2A/CD94 interaction than those for the HLA-G*01:03/NKG2A/CD94 and HLA-G*01:01/NKG2A/CD94 interactions. Moreover, no engagement was observed between the activating receptor NKG2C/CD94 and HLA-G. HLA-G allelic product dependent binding of NKG2A/CD94 could result from the single amino acid residue in the HLA-G*01:01 heavy chain differing from HLA-G*01:04 (p.110L > I) and HLA-G*01:03 (p.31T > S), and these different residues may affect the peptide repertoire and receptor recognition (111). Consequently, different biological function modulation could be expected for the different allelic HLA-G molecules on the immune cells expressing NKG2A/CD94.

Other receptors are the glycosylphosphatidylinositol-anchored receptor CD160, which is expressed on activated endothelial cells, CD16^+^CD56^dim^ NK cells, and CD8^+^ T cells (112, 113). CD160 signaling depends on adapter proteins, such as the phosphoinositide-3 kinase, to activate endothelial cell migration and angiogenesis, and immune cells, such as cytokine releasing and target cell lyses (112, 114). Moreover, the cytotoxic T cell surface marker CD8 is reported to interact with the HLA-G protein, which can induce apoptosis of the CD8^+^ T cells and CD56^+^CD8^+^ NKT cells through the Fas/FasL pathway (115).

## Conclusions and Perspectives

In most scenarios, HLA-G/ILTs interaction promotes cancer cells to escape immune surveillance and anti-tumor immunity (97, 116). Interference or blockade of HLA-G/ILTs interaction can restore host anti-tumor immune responses, which providing a strong rationale and opportunities to develop HLA-G/ILTs-targeted ICIs for solid cancer immunotherapy. Indeed, early clinical trials based on this immune checkpoint is being launched for different advanced solid cancer treatment^2^. However, many challenges remain to be addressed.

First, multiple identified HLA-G isoforms and more can be expected, which have distinct extracellular domain(s) for each of them. This information indicates that biological function of different isoforms is diverse. Being lack of isoform-specific antibodies, their tumor tissue expression characteristics and clinical significance is unclear. Currently available mAb 4H84, which probes all isoforms containing α1 domain, can’t distinguish either a distinct isoform or combination of different isoforms expressed on tumor tissues. Novel isoforms without α1 domain can’t be detected with any available mAbs which can be ignored in tumor tissues. These findings sharply compounded the clinical relevance of HLA-G in tumor patients (62). Second, being lack of international validated or recommended assay protocols, both performance and cut-off points are diverse and unconcordance in interpretation of HLA-G expression across studies is rather common (117). Third, being the landscape of HLA-G isoform expression in tumor tissues can’t be specified, application of HLA-G/ILTs-targeted ICIs for cancer treatment can be aimless. Furthermore, HLA-G/ILTs engagement is isoform dependent, where HLA-G1 and HLA-G5 binds ILT-2, and HLA-G1, -G2, -G4, -G5, and HLA-G6 binds ILT-4 (118). Differential expression of HLA-G isoforms does exist in tumor cells whereas the underlying mechanisms are yet to be explored. In this scenario, patient individualized landscape and degree of HLA-G isoform expression should be defined before therapy due to which can tremendously affect clinical benefits of HLA-G-based solid cancer immunotherapy.

In summary, exploration of more reliable and HLA-G isoform-specific antibodies, implementation of international community validated HLA-G detection protocols, deeper insight of patient individualized landscape of HLA-G expression, and development of isoform matched both HLA-G and ILT-2/-4 blocking antibodies, are future directions warranted for the HLA-G/ILTs-targeted solid cancer immunotherapy. Despite these challenges, owing to HLA-G expression is restrict to malignant tissues, and HLA-G/ILTs signaling is involved in a broader spectrum of immune responses than CTLA-4/B7 and PD-1/PD-L1 does, the clinical effects of the immune checkpoint HLA-G/ILTs is optimistic.

## Author Contributions

Conceptualization, data curation, funding acquisition, writing —original draft, review and editing: W-HY and AL. Both authors contributed to the article and approved the submitted version.

## Funding

This work was supported by grants from Science and Technology Bureau of Taizhou (1901ky01; 1901ky04).

## Conflict of Interest

The authors declare that the research was conducted in the absence of any commercial or financial relationships that could be construed as a potential conflict of interest.

## Supplementary Material

Supplementary Figure 1 CLUSTALO sequence alignment either among or between full-length HLA-G, HLA-F, and HLA-E molecules. Results of sequence alignment **(A)** among full length of HLA-G, HLA-F, and HLA-E molecules. **(B)** Between full length of HLA-G and HLA-F molecules. **(C)** Between full length of HLA-E and HLA-F molecules. **(D)** Between full length of HLA-E and HLA-G molecules.
