# Supplementary material for: HLA-G/ILTs Targeted Solid Cancer Immunotherapy: Opportunities and Challenges
Source: Front Immunol. 2021 Jun 30;12:698677. doi: 10.3389/fimmu.2021.698677 (PMC8278316; doi:10.3389/fimmu.2021.698677)
Supplement: Supplementary Figure 1 — CLUSTALO sequence alignment either among or between full-length HLA-G, HLA-F, and HLA-E molecules. Results of sequence alignment (A) among full length of HLA-G, HLA-F, and HLA-E molecules. (B) Between full length of HLA-G and HLA-F molecules. (C) Between full length of HLA-E and HLA-F molecules. (D) Between full length of HLA-E and HLA-G molecules. [file DataSheet_1.pdf]

## Suppl. Figure 1.

**(A)**

```
CLUSTAL O(1.2.4) multiple sequence alignment

SP|P30511|HLAF_HUMAN ---MAPRSLLLLLSGALALTDTWAGSHSLRYFSTAVSRPGRGEPRYIAVEYVDDTQFLRF 57
SP|P17693|HLAG_HUMAN MVVMAPRTLFLLLSGALTLTETWAGSHSMRYFSAAVSRPGRGEPRFIAMGYVDDTQFVRF 60
SP|P13747|HLAE_HUMAN ---MVDGTLLLLLSEALALTQTWAGSHSLKYFHTSVSRPGRGEPRFISVGYVDDTQFVRF 57
                        *.  :*:**** **:**:*******::** ::**********:*:: *******:**
SP|P30511|HLAF_HUMAN DSDAAIPRMEPREPWVEQEGPQYWEWTTGYAKANAQTDRVALRNLLRRYNQSEAGSHTLQ 117
SP|P17693|HLAG_HUMAN DSDSACPRMEPRAPWVEQEGPEYWEEETRNTKAHAQTDRMNLQTLRGYYNQSEASSHTLQ 120
SP|P13747|HLAE_HUMAN DNDAASPRMVPRAPWMEQEGSEYWDRETRSARDTAQIFRVNLRTLRGYYNQSEAGSHTLQ 117
                     *.*:* *** ** **:**** :**:  *  ::  **  *: *:.*   ******.*****
SP|P30511|HLAF_HUMAN GMNGCDMGPDGRLLRGYHQHAYDGKDYISLNEDLRSWTAADTVAQITQRFYEAEEYAEEF 177
SP|P17693|HLAG_HUMAN WMIGCDLGSDGRLLRGYEQYAYDGKDYLALNEDLRSWTAADTAAQISKRKCEAANVAEQR 180
SP|P13747|HLAE_HUMAN WMHGCELGPDGRFLRGYEQFAYDGKDYLTLNEDLRSWTAVDTAAQISEQKSNDASEAEHQ 177
                      * **::* ***:****.*.*******::**********.**.***:::   :  .  **.
SP|P30511|HLAF_HUMAN RTYLEGECLELLRRYLENGKETLQRADPPKAHVAHHPISDHEATLRCWALGFYPAEITLT 237
SP|P17693|HLAG_HUMAN RAYLEGTCVEWLHRYLENGKEMLQRADPPKTHVTHHPVFDYEATLRCWALGFYPAEIILT 240
SP|P13747|HLAE_HUMAN RAYLEDTCVEWLHKYLEKGKETLLHLEPPKTHVTHHPISDHEATLRCWALGFYPAEITLT 237
                     *:***. *:* *::***:*** * : :***:**:***: *:**************** **
SP|P30511|HLAF_HUMAN WQRDGEEQTQDTELVETRPAGDGTFQKWAAVVVPPGEEQRYTCHVQHEGLPQPLILRWEQ 297
SP|P17693|HLAG_HUMAN WQRDGEDQTQDVELVETRPAGDGTFQKWAAVVVPSGEEQRYTCHVQHEGLPEPLMLRWKQ 300
SP|P13747|HLAE_HUMAN WQQDGEGHTQDTELVETRPAGDGTFQKWAAVVVPSGEEQRYTCHVQHEGLPEPVTLRWKP 297
                     **:*** :***.********************** ****************:*: ***:
SP|P30511|HLAF_HUMAN SPQPTIPIVGIVAGLVVLGAVVTGAVVAAVMWRKKSSDRNRGSYSQAAV----------- 346
SP|P17693|HLAG_HUMAN SSLPTIPIMGIVAGLVVLAAVVTGAAVAAVLWRKKSSD---------------------- 338
SP|P13747|HLAE_HUMAN ASQPTIPIVGIIAGLVLLGSVVSGAVVAAVIWRKKSSGGKGGSYSKAEWSDSAQGSESHS 357
                     :  *****:**:****:*.:**:**.****:******.
SP|P30511|HLAF_HUMAN -
SP|P17693|HLAG_HUMAN -
SP|P13747|HLAE_HUMAN L 358
```

| | |
|---|---|
| Date of job execution | 2021-05-23 |
| Job identifier | A2021052372FEB3358BE035486EE75ADE9E917725007A9BH |
| Running time | 14.8 seconds |
| Identical positions | 225 |
| Identity | 62.327% |
| Similar positions | 67 |
| Program | CLUSTALO |

**(B)**

```
CLUSTAL O(1.2.4) multiple sequence alignment


SP|P30511|HLAF_HUMAN ---MAPRSLLLLLSGALALTDTWAGSHSLRYFSTAVSRPGRGEPRYIAVEYVDDTQFLRF 57
SP|P17693|HLAG_HUMAN MVVMAPRTLFLLLSGALTLTETWAGSHSMRYFSAAVSRPGRGEPRFIAMGYVDDTQFVRF 60
                        ****:*:*******:**:*******:****:***********:**: *******:**

SP|P30511|HLAF_HUMAN DSDAAIPRMEPREPWVEQEGPQYWEWTTGYAKANAQTDRVALRNLLRRYNQSEAGSHTLQ 117
SP|P17693|HLAG_HUMAN DSDSACPRMEPRAPWVEQEGPEYWEEETRNTKAHAQTDRMNLQTLRGYYNQSEASSHTLQ 120
                     ***:* ****** ********:***  *  :**:*****: *:.*   ******.*****

SP|P30511|HLAF_HUMAN GMNGCDMGPDGRLLRGYHQHAYDGKDYISLNEDLRSWTAADTVAQITQRFYEAEEYAEEF 177
SP|P17693|HLAG_HUMAN WMIGCDLGSDGRLLRGYEQYAYDGKDYLALNEDLRSWTAADTAAQISKRKCEAANVAEQR 180
                      * ***:* ********.*:*******::*************.***::*  ** : **:

SP|P30511|HLAF_HUMAN RTYLEGECLELLRRYLENGKETLQRADPPKAHVAHHPISDHEATLRCWALGFYPAEITLT 237
SP|P17693|HLAG_HUMAN RAYLEGTCVEWLHRYLENGKEMLQRADPPKTHVTHHPVFDYEATLRCWALGFYPAEIILT 240
                     *:**** *:* *:******** ********:**:***: *:**************** **

SP|P30511|HLAF_HUMAN WQRDGEEQTQDTELVETRPAGDGTFQKWAAVVVPPGEEQRYTCHVQHEGLPQPLILRWEQ 297
SP|P17693|HLAG_HUMAN WQRDGEDQTQDVELVETRPAGDGTFQKWAAVVVPSGEEQRYTCHVQHEGLPEPLMLRWKQ 300
                     ******:****.********************** ****************:**:***:*

SP|P30511|HLAF_HUMAN SPQPTIPIVGIVAGLVVLGAVVTGAVVAAVMWRKKSSDRNRGSYSQAAV 346
SP|P17693|HLAG_HUMAN SSLPTIPIMGIVAGLVVLAAVVTGAAVAAVLWRKKSSD----------- 338
                     *  *****:*********.******.****:*******
```

| | |
|---|---|
| Date of job execution | 2021-05-23 |
| Job identifier | A2021052372FEB3358BE035486EE75ADE9E917725007AA1N |
| Running time | 14.4 seconds |
| Identical positions | 265 |
| Identity | 75.931% |
| Similar positions | 43 |
| Program | CLUSTALO |

**(C)**

```
CLUSTAL O(1.2.4) multiple sequence alignment


SP|P30511|HLAF_HUMAN MAPRSLLLLLSGALALTDTWAGSHSLRYFSTAVSRPGRGEPRYIAVEYVDDTQFLRFDSD 60
SP|P13747|HLAE_HUMAN MVDGTLLLLLSEALALTQTWAGSHSLKYFHTSVSRPGRGEPRFISVGYVDDTQFVRFDND 60
                     *.  :****** *****:********:** *:**********:*:* *******:***.*
SP|P30511|HLAF_HUMAN AAIPRMEPREPWVEQEGPQYWEWTTGYAKANAQTDRVALRNLLRRYNQSEAGSHTLQGMN 120
SP|P13747|HLAE_HUMAN AASPRMVPRAPWMEQEGSEYWDRETRSARDTAQIFRVNLRTLRGYYNQSEAGSHTLQWMH 120
                     ** *** ** **:**** :**:  *  *: .**  ** **.*   ************ *:
SP|P30511|HLAF_HUMAN GCDMGPDGRLLRGYHQHAYDGKDYISLNEDLRSWTAADTVAQITQRFYEAEEYAEEFRTY 180
SP|P13747|HLAE_HUMAN GCELGPDGRFLRGYEQFAYDGKDYLTLNEDLRSWTAVDTAAQISEQKSNDASEAEHQRAY 180
                     **::*****:****.*.*******::**********.**.***:::  :  . **. *:*
SP|P30511|HLAF_HUMAN LEGECLELLRRYLENGKETLQRADPPKAHVAHHPISDHEATLRCWALGFYPAEITLTWQR 240
SP|P13747|HLAE_HUMAN LEDTCVEWLHKYLEKGKETLLHLEPPKTHVTHHPISDHEATLRCWALGFYPAEITLTWQQ 240
                     **. *:* *::***:***** : :***:**:****************************:
SP|P30511|HLAF_HUMAN DGEEQTQDTELVETRPAGDGTFQKWAAVVVPPGEEQRYTCHVQHEGLPQPLILRWEQSPQ 300
SP|P13747|HLAE_HUMAN DGEGHTQDTELVETRPAGDGTFQKWAAVVVPSGEEQRYTCHVQHEGLPEPVTLRWKPASQ 300
                     *** :************************** ****************:*: ***: : *
SP|P30511|HLAF_HUMAN PTIPIVGIVAGLVVLGAVVTGAVVAAVMWRKKSSDRNRGSYSQAAV------------ 346
SP|P13747|HLAE_HUMAN PTIPIVGIIAGLVLLGSVVSGAVVAAVIWRKKSSGGKGGSYSKAEWSDSAQGSESHSL 358
                     ********:****:**:**:*******:******. : ****:*
```

| | |
|---|---|
| Date of job execution | 2021-05-23 |
| Job identifier | A202105235C475328CEF75220C360D524E9D456CE010A8CB |
| Running time | 28.7 seconds |
| Identical positions | 251 |
| Identity | 70.112% |
| Similar positions | 55 |
| Program | CLUSTALO |

**(D)**

```
CLUSTAL O(1.2.4) multiple sequence alignment

SP|P17693|HLAG_HUMAN MVVMAPRTLFLLLSGALTLTETWAGSHSMRYFSAAVSRPGRGEPRFIAMGYVDDTQFVRF 60
SP|P13747|HLAE_HUMAN ---MVDGTLLLLLSEALALTQTWAGSHSLKYFHTSVSRPGRGEPRFISVGYVDDTQFVRF 57
                        *.  **:**** **:**:*******::** ::************::***********
SP|P17693|HLAG_HUMAN DSDSACPRMEPRAPWVEQEGPEYWEEETRNTKAHAQTDRMNLQTLRGYYNQSEASSHTLQ 120
SP|P13747|HLAE_HUMAN DNDAASPRMVPRAPWMEQEGSEYWDRETRSARDTAQIFRVNLRTLRGYYNQSEAGSHTLQ 117
                     *.*:*.*** *****:**** ***:.***.::   **   *:**:***********.*****
SP|P17693|HLAG_HUMAN WMIGCDLGSDGRLLRGYEQYAYDGKDYLALNEDLRSWTAADTAAQISKRKCEAANVAEQR 180
SP|P13747|HLAE_HUMAN WMHGCELGPDGRFLRGYEQFAYDGKDYLTLNEDLRSWTAVDTAAQISEQKSNDASEAEHQ 177
                     ** **:** ***:******:********:**********.*******::*.: *.  **::
SP|P17693|HLAG_HUMAN RAYLEGTCVEWLHRYLENGKEMLQRADPPKTHVTHHPVFDYEATLRCWALGFYPAEIILT 240
SP|P13747|HLAE_HUMAN RAYLEDTCVEWLHKYLEKGKETLLHLEPPKTHVTHHPISDHEATLRCWALGFYPAEITLT 237
                     *****.*******:***:*** * : :**********: *:**************** **
SP|P17693|HLAG_HUMAN WQRDGEDQTQDVELVETRPAGDGTFQKWAAVVVPSGEEQRYTCHVQHEGLPEPLMLRWKQ 300
SP|P13747|HLAE_HUMAN WQQDGEGHTQDTELVETRPAGDGTFQKWAAVVVPSGEEQRYTCHVQHEGLPEPVTLRWKP 297
                     **:***.:***.*****************************************: ****
SP|P17693|HLAG_HUMAN SSLPTIPIMGIVAGLVVLAAVVTGAAVAAVLWRKKSSD---------------------- 338
SP|P13747|HLAE_HUMAN ASQPTIPIVGIIAGLVLLGSVVSGAVVAAVIWRKKSSGGKGGSYSKAEWSDSAQGSESHS 357
                     :* *****:**:****:*.:**:**.****:******.
SP|P17693|HLAG_HUMAN -
SP|P13747|HLAE_HUMAN L 358
```

| | |
|---|---|
| Date of job execution | 2021-05-23 |
| Job identifier | A2021052372FEB3358BE035486EE75ADE9E917725007AA3Q |
| Running time | 14.4 seconds |
| Identical positions | 257 |
| Identity | 71.191% |
| Similar positions | 56 |
| Program | CLUSTALO |

**'*'** indicates positions which have a single, fully conserved residue

**':'** indicates that one of the following 'strong' groups is fully conserved

**'.'** indicates that one of the following 'weaker' groups is fully conserved
